# Enzymatic Hydrolysis as an Effective Method for Obtaining Wheat Gluten Hydrolysates Combining Beneficial Functional Properties with Health-Promoting Potential

**DOI:** 10.3390/molecules29184407

**Published:** 2024-09-16

**Authors:** Magdalena Mika, Agnieszka Wikiera

**Affiliations:** 1 Department of Biotechnology and General Food Technology, Faculty of Food Technology, Agricultural University of Krakow, 31-120 Krakow, Poland; 2 Department of Medical Physiology, Faculty of Health Sciences, Jagiellonian University Medical College, 31-008 Krakow, Poland; agnieszka.wikiera@uj.edu.pl

**Keywords:** wheat gluten, proteases, protein hydrolysates, functional properties, biological activity

## Abstract

The byproduct from wheat starch production contains approximately 70% gluten (WG) and is an inexpensive but demanding protein raw material for the food industry. This study attempted to determine the optimal hydrolysis conditions for such raw material to obtain peptides combining beneficial functional characteristics with health-promoting activity. The proteases Bromelain, Alcalase, Flavourzyme, and a protease from *A. saitoi* were used for hydrolysis. It was shown that the tested proteases differ both in terms of the effective hydrolysis conditions of gluten and the profile of the released hydrolysates. Bromelain was particularly effective in converting gluten into peptides, combining beneficial health and functional properties. It achieved maximum activity (189 U/g) against WG at pH 6 and 60 °C, and the best-balanced peptides in terms of desired properties were released at a dose of 2.5 U/g. These peptides were free from most allergenic epitopes, effectively inhibited ACE, and, at 0.34 g, were equivalent to the approved dose of BHT. Their emulsifying activity was higher than that of gluten, and the foaming formation and stabilization potential exceeded that of ovalbumin by 10% and 19%, respectively. It seems that Bromelain-released WG hydrolysates are a promising candidate for a safe fat stabilizer and egg white substitute.

## 1. Introduction

Gluten, the principal storage protein of cereal grains, plays a crucial role in determining the quality of the flour and, subsequently, the dough obtained from it. It is also a primary byproduct of industrial starch and bioethanol production [1], making it readily available and inexpensive as a protein source for the food industry. Wheat gluten comprises two fractions distinguished by differences in structure and electrophoretic mobility: gliadins and glutenins. Gliadins are predominantly monomeric proteins, characterized by the presence of specific amino acid sequences and molecular weights ranging from 31.8 to 50.9 kDa. On the other hand, glutenins are polymeric proteins cross-linked by disulfide bonds (S-S), which make many of them among the largest proteins in nature [2]. Regardless of the fraction, wheat gluten is a protein with an atypical amino acid composition that defines its properties and functions. From 32 to 57% of this polymer comprises glutamine (Q), which contributes an amino group in the side chain (acting as a nitrogen reservoir). Proline (P) accounts for 11–29%, forming atypical peptide bonds that shield gluten from enzymatic hydrolysis. Finally, up to 28% consists of nonpolar amino acids V, L, I, and F, responsible for the protein’s hydrophobicity, thus safeguarding it from leaking out of the grain during germination [3]. This composition has significant implications for potential applications in food technology. Firstly, gluten is poorly soluble in water. Secondly, it undergoes only limited hydrolysis in the gastrointestinal tract. Thirdly, it contains amino acid sequences with allergenic potential, such as PSQQ, QQQP, QQPY, or QPYP, contributing to the development of celiac disease in individuals genetically predisposed, as well as non-celiac gluten sensitivity, atopic dermatitis, Baker’s asthma, and wheat-dependent exercise-induced allergy [2]. One of the widely tested solutions aimed at addressing the drawbacks of gluten is its limited enzymatic hydrolysis. For this purpose, both endo- and exoproteases are employed. Typically, these are inexpensive commercial preparations possessing more than one activity, including activity towards bonds formed with the involvement of P or other amino acids with nonpolar groups [4,5]. An example of such a product is Flavourzyme, derived from *Aspergillus oryzae.* It contains seven different proteases, including three endopeptidases (neutral NP1, neutral NP2, alkaline ALP1), two leucine aminopeptidases (LAPA and LAP2), and two dipeptidyl peptidases (DPP4 and DPP5) [6], thus ensuring a high degree of gluten hydrolysis. Products with a lower diversity of proteases are also being tested, which is expected to promote better retention of the structural properties of the hydrolysate. One such preparation is Alcalase, derived from *Bacillus licheniformis*, which is a serine endopeptidase characterized by its ability to hydrolyze bonds occupied at the P1 position by E, M, L, Y, K, or Q [7]. This preparation exhibits its highest activity in an alkaline environment. A similar product, but showing optimal activity in acidic pH, is the endoprotease from *Aspergillus saitoi*. It prefers the hydrolysis of bonds where the donor of the carboxyl group is a hydrophobic amino acid such as F, Y, L, or I [8]. Another tool is Bromelain, produced from pineapple processing waste [5]. This preparation contains four isoforms of thiol endopeptidases, preferring the hydrolysis of bonds formed by G, A, L, or Q. It also contains other proteinaceous and non-proteinaceous factors contributing to its multifaceted, health-promoting effects, and it enjoys widespread recognition in the phytotherapeutics market [9,10]. To obtain wheat gluten hydrolysates (WGHs) with desired characteristics, these and other proteases are tested not only individually but also in the form of multi-activity cocktails that act on both native substrates and variously denatured substrates [11,12]. However, despite extensive efforts, the project based on the zero-waste concept of utilizing wheat gluten for the production of hydrolysates that combine health-promoting benefits with sought-after functional properties in food technology still remains partially unresolved.

A detailed literature review on the utilization of gluten for protein hydrolysates reveals that in the vast majority of studies, proteases were treated somewhat like mechanical tools, neglecting their significant adaptability in response to changes in the environment. As a result, the conditions of their activity were not optimized, and their activity against non-typical substrates was not determined [11,13,14,15]. When this was performed, instead of using gluten, other proteins [16] or peptides [17] were often employed. In this work, we aim to change that. It has long been known that the effectiveness, and even the direction of action, of enzymes, including proteases, depends on pH. It is also clear that a given enzyme, acting on different substrates, may require a different concentration of hydrogen ions, which not only ensures optimal ionization of functional groups in the active center of the catalyst but also affects the ionization of the substrate [18,19]. It is also known that both enzyme activity and the availability of their substrates, especially those of a protein nature, are strictly dependent on temperature [20] and the E/S ratio [21]. In the case of a substrate such as gluten, temperature may induce, for example, conformational changes [22] that affect the availability of peptide bonds for proteases. On the other hand, an excessive amount of hydrolase relative to its substrate promotes so-called reversed proteolysis, significantly limiting the efficiency of hydrolysis [21]. In this work, we present the possibilities that full optimization of reaction conditions offers in the field of obtaining WGHs for four commercial preparations: Alcalase, Bromelain, Flavourzyme, and proteases from *Aspergillus saitoi*. The selection of these preparations was determined by their specificity towards different peptide bonds, ensuring a wide variety of peptides in terms of mass and amino acid composition and, consequently, their properties. Their availability and origin, ensuring low costs of obtaining WGHs useful in designing food for various consumers, including vegetarians and vegans, were also important factors. In this study, we identify the effective doses of each enzymatic preparation against WG and provide a comprehensive characterization of the hydrolysates obtained using them. We examine the allergenic potential of the resulting WGHs and their suitability as substances enabling the creation and stabilization of foams and emulsions. We also verify their usefulness as substances that stabilize fats and inhibit angiotensin-converting enzymes.

## 2. Results and Discussion

### 2.1. Optimization of Enzymatic Gluten Hydrolysis Conditions

**Figure 1:**
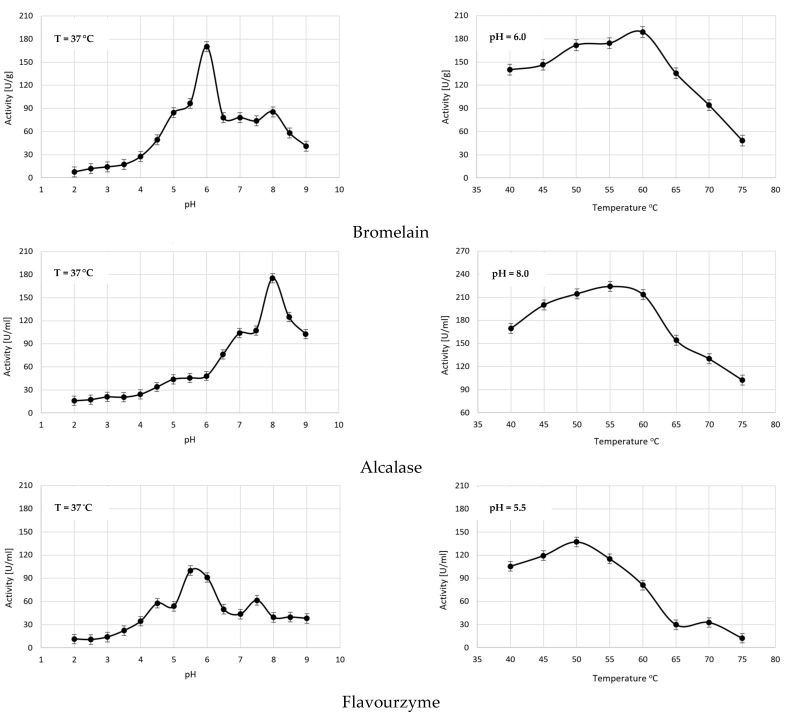

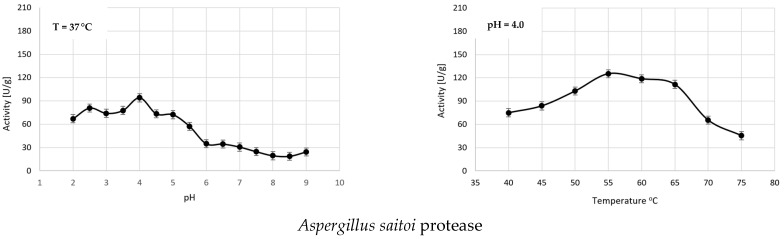
The influence of pH and temperature on the ability of proteolytic preparations—Bromelain, Alcalase, Flavourzyme, and *A. saitoi* protease—to WG hydrolyze. All data represent the means and standard deviations (*n*  =  4). Enzymatic preparations were used at an E:S ratio of 1:250 (*w*/*v*). The optimal pH of the enzymatic preparations was assessed at a temperature of 37 °C with a reaction time of 10 min. The optimal hydrolysis temperature was determined after considering the optimal pH by conducting the reaction for 45 min.

The influence of environmental acidity on the capability of four proteolytic preparations—Alcalase, Flavourzyme, Bromelain, and *A. saitoi* protease—to hydrolyze WG was investigated within a pH range of 2 to 9 at a temperature of 37 °C. The results are presented in Figure 1. It was observed that, in response to the tested changes in H^+^ concentration, the examined enzymatic preparations exhibited a 5-fold (*A. saitoi* protease) to 20-fold (Bromelain) change in activity levels against WG, demonstrating very narrow pH optima. The optimal pH values for gluten depolymerization by Alcalase, Flavourzyme, Bromelain, and *A. saitoi* protease were 8.0, 5.5, 6.0, and 4.0, respectively. Meanwhile, in studies related to the use of Alcalase in WGH production, pH values of around 8.5 [14,23], 9.0 [24], or 7.0 [25] are commonly used. Our results clearly indicate that the activity of Alcalase against gluten in such systems is up to 40% lower (at pH 8.5) and even 70% lower (at pH 7 and 9) than what this preparation can achieve at pH 8. Similar discrepancies also apply to the Flavourzyme preparation. In this case, the pH values ranging from 6.8 to 7.5 [16,25,26] are most commonly used for obtaining WGHs. However, according to the relationship shown in Figure 1, the Flavourzyme activity against gluten reaches a maximum at pH 5.5 and is up to 120% higher than at pH 7 and 60% higher than at pH 7.5. Bromelain is most commonly used for the hydrolysis of wheat gluten at pH 6 [27], which is consistent with the optimization results obtained in our studies. On the other hand, in the studies by Bradauskiene et al. [5], pH optimization for the hydrolysis of gluten in wheat bran was conducted, and it was shown that the optimal pH for this product is 4. These results indicate that the optimal pH is influenced not only by the type of protease and the protein being hydrolyzed but also by the type of matrix. For this extensively utilized enzymatic preparation, detailed data regarding optimal pH values for its activity depending on the type of hydrolyzed substrate can be found. The differences are substantial. It turns out the Bromelain most effectively hydrolyzes hemoglobin at pH 2.8, sodium caseinate at pH 6.5, and the synthetic peptide benzoylo-FVR-PNA only at pH 8.0 [20].

Second only to pH, temperature is a significantly influential factor in the progression of any reaction. In enzymatic processes, it affects not only the apoenzyme but also the substrates and individual reaction steps. Protein substrates undergo denaturation in response to increasing temperature, which usually enhances their susceptibility to hydrolysis, although not always. In the case of wheat gluten temperatures above 55 °C may cause the formation of additional S-S bridges [28], which decrease its availability to the enzyme. Consequently, one enzyme catalyzing transformations of different proteins may have different temperature requirements for each of them; for example, fruit Bromelain hydrolyzes hemoglobin most effectively at 37 °C but casein at 60 °C [20]. The determination of the optimal temperature for the hydrolysis of WG by Alcalase, Flavourzyme, Bromelain, and *A. saitoi* protease was carried out at the pH previously considered to be the best for each of the preparations. The reaction time, as a parameter significantly affecting the observed temperature optimum, was increased to 45 min. Temperatures were analyzed in the range from 40 to 75 °C, and the obtained results are presented in Figure 1. Among the tested proteases, Flavourzyme was the most temperature-sensitive; its activity ranged from 12 to 137 U/mL, reaching a maximum of 50 °C. For Alcalase and *A. saitoi* protease, the highest activity was observed at 55 °C, while for Bromelain, it was 60 °C. Comparing the determined optima for proteases with temperatures previously used for gluten hydrolysis, a greater alignment can be observed compared to the situation with pH. According to literature data, gluten hydrolysis using Alcalase was most commonly performed at 50–60 °C [13,14,16,23,25]. Our research indicates that differences in enzyme activity in this range are small and reach about 5%. Flavourzyme is typically used for gluten hydrolysis at 50 °C [16,25], although studies proposing 37 °C can also be found [26], resulting in a decrease in activity of approximately 23%. Meanwhile, for Bromelain, the most commonly chosen temperature is 50 °C [5,27], or even 45 °C [5], although according to our data, under these conditions, this protease exhibits 91% and 77% of its maximum activity, respectively.

In conclusion, each of the tested proteases required different pH and temperature conditions to achieve maximum hydrolytic activity towards GW, and these parameters, as cited in the literature, did not always align with the optimal conditions for hydrolyzing other proteins using the same preparations. This means that the concepts of “optimal temperature” and “optimal pH” are specific to a particular reaction rather than to a specific enzyme, which is why the process of optimizing the conditions for a given enzymatic reaction is so important and should not be overlooked.

### 2.2. Determination of the Maximum Activity and Optimal Dose of Proteases for WG Hydrolysis

The tested proteolytic preparations used for wheat gluten hydrolysis under their optimal conditions differed significantly in their maximum activity, defined as the amount of µmol of amino groups released per minute from gluten. The results are presented in Table 1.

Among the proteases studied, Alcalase exhibited the highest activity against WG. The activities of Bromelain, Flavourzyme, and *A. saitoi* protease were approximately 16%, 39%, and 44% lower, respectively. According to the review by Tacias-Pascacio et al. [7], Alcalase is known for its exceptionally high activity and broad spectrum of recognized amino acids, enabling it to effectively hydrolyze almost all protein substrates, including gluten.

The maximum activity of the proteases against WG as a substrate was used to determine the range of doses at which these preparations should act on the substrate with the desired efficiency during a four-hour hydrolysis. Assuming an average molecular mass of the amino acids in wheat gluten of 137.9 g/mol, it was calculated that a dose of approximately 30 U is required for the complete hydrolysis of 1 g of WG in 4 h. However, considering that endoproteases hydrolyze at most 10–35% of the peptide bonds present in the protein and that the Flavourzyme preparation possesses significant exoprotease activity, the tested range of E/S doses was limited to between 2.5 and 25 U/g of WG. The proper selection of enzyme dosage in the hydrolysis process can be a crucial step, as it allows for achieving a specific degree of substrate depolymerization. However, more enzymes do not always result in deeper hydrolysis. Proteases, particularly those like pepsin, trypsin, and papain, are capable of catalyzing so-called reversed proteolysis and transpeptidation, which lead to the ligation of previously released peptides [21].

### 2.3. The Effect of Protease Dose on the Gluten Hydrolysis Degree and Protein Recovery in WGHs

The degree of hydrolysis (DH%) and protein recovery (PR%) for gluten converted for 4 h with different doses of preparations: Alcalase, Bromelain, Flavourzyme, and protease from *A. saitoi* are shown in Table 2.

DH and PR are parameters that allow for the assessment of the efficiency and cost-effectiveness of the hydrolysis process. The solubility of WG ranges from 2.8% at pH 7 to 10.6% at pH 2, but hydrolysis of even 5% of peptide bonds causes it to increase by up to 71% [16]. A high PR indicates minimal losses in the applied technology for WGH production and, given the low cost of raw materials, can be decisive for its commercial use. In our studies, the degree of WG depolymerization in the presence of all proteases increased with their dosage without reaching a plateau. For 2.5 U/g of gluten, products with DH ranging from 6.3% to 27.5% were obtained, and a tenfold increase in enzyme dosage resulted in an increase in DH to levels between 12.9% and 64.2%.

The proteases’ ability to deeply convert WG decreased in the following sequence: Flavourzyme > Alcalase > Bromelain > *A. saitoi* protease. In the studied conditions, mathematical models were also developed to determine the degree of hydrolysis depending on the enzyme activity used [U per gram of dry WG], respectively for Bromelain (1) y = 0.0046x^3^ − 0.2105x^2^ + 3.364x + 0.3381, Alcalase (2) y = 0.0086x^3^ − 0.3762x^2^ + 5.286x + 1.4843, R^2^ = 0.962, Flavourzyme (3) y = 0.0164x^3^ − 0.7539x^2^ + 11.101x + 1.8896, R^2^ = 0.983, *A. saitoi* protease (4) y = 0.0039x^3^ − 0.173x^2^ + 2.4073x + 0.474, R^2^ = 0.983. It is important to emphasize that the maximum values of the degree of hydrolysis of native gluten shown in Table 2 were higher than those reported by other researchers using the same proteases. This is probably a consequence of the optimization made in pH and temperature for the reaction under study. In the case of Alcalase acting on WG, Kong et al. [14] reported a DH of 15.8%, and He et al. [16] reported a DH of 16.8%, while in our study, this value reached even 33.2%. For Bromelain, Banerjee et al. [27] achieved a DH of 10%, whereas we observed a DH of 25.2%. Finally, for WGHs obtained in the presence of Flavourzyme, Zhao et al. [11] reported a DH of 7.5%, Kim [12] reported 23.7%, and Merz et al. [26] reported 42.5%. In the experiment described here, the DH reached 64.2%. It seems that with such a high ability of the Flavourzyme protease to hydrolyze WG very deeply, the WGHs obtained in this way could be useful as a spice ingredient. It is known from the literature that gluten hydrolysis products with DH above 30% acquire taste and smell [12]. It is also known that bitter peptides, which WG may be the source of due to the high content of nonpolar amino acids and glutamine, usually have masses from 500 to 1500 Da [29], so it can be assumed that in WGHs with DH of 64%, they will be poorly represented.

In contrast, PR increased with the dose of the preparations thereby increasing with DH of the hydrolysates obtained under the influence of a given protease (Table 2). In WGHs obtained under the influence of Flavourzyme, Alcalase, and Bromelain, the maximum PR was similar, ranging from a satisfactory 81.2% for the lowest doses of these preparations to a very high 92.8% for the highest doses. However, for a PR of 92%, Flavourzyme had to hydrolyze 57% of the peptide bonds of gluten, while Alcalase was 33% and Bromelain only 25%. This result confirms the different nature of these proteases, as described in the introduction. It clearly indicates the cooperation of exo- and endoproteases present in Flavourzyme and the greater selectivity of Bromelain towards the targeted bonds compared to Alcalase.

### 2.4. The Effect of Dose and Type of Protease on the Allergenic Potential of the Obtained WGHs 

**Figure 2:**
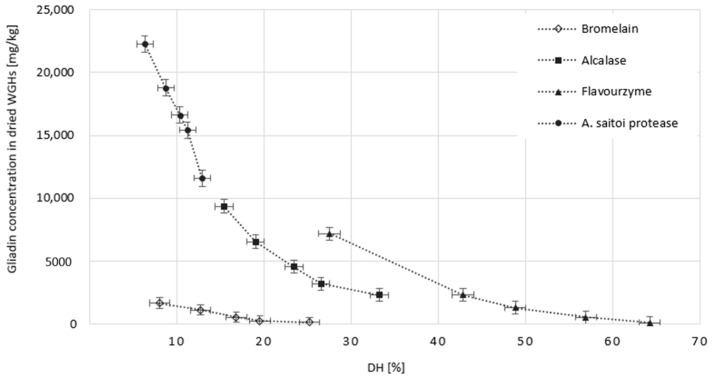
The influence of DH on allergenic fraction content in dried WGHs obtained using Bromelain, Alcalase, Flavourzyme, and *A. saitoi* protease. All data represent the means and standard deviations (*n* = 4).

One of the fundamental issues associated with the use of WG and its hydrolysates in food processing is the presence of epitopes that interact with the immune system and induce the development of celiac disease or allergies. The hydrolysis of peptide bonds within these sequences can reduce or even eliminate their allergenicity. The presence of undesirable epitopes in WGHs was estimated using the Ridascreen^®^ ELISA (R-Biopharm, Darmstadt, Germany) and expressed as mg of gliadin per kg of WGH dry matter. The results in relation to the DH are presented in Figure 2.

According to the FAO-WHO Codex Alimentarius, if the gluten-allergenic fraction content in a product, as determined by the Ridascreen^®^ test, does not exceed 20 mg/kg, the product is considered gluten-free. If the content falls within the range of 20–100 mg/kg, the product is classified as low-gluten. All tested proteases exhibited the ability to hydrolyze allergenic gluten sequences. This ability was dependent on the type of preparation but consistently increased with its dosage and thus with the degree of gluten hydrolysis. Bromelain and Flavourzyme showed the greatest affinity for immunotoxic epitopes. However, only Bromelain significantly reduced the allergenicity of the protein by hydrolyzing just 8% of its peptide bonds. In contrast, to achieve a product with similar allergenicity, Flavourzyme needed to degrade approximately 48% of the peptide bonds present in gluten. The potential use of proteases to reduce gluten allergenicity has been noted many times. However, this typically involves very deep protein hydrolysis, which eliminates its valuable functional properties [5,30,31]. Merz et al. [26], in their search for a convenient tool to generate non-allergenic WGHs, proposed the use of Flavourzyme, Bioprase, and Thermoase preparations. However, only Flavourzyme demonstrated high efficacy against undesirable epitopes, but at the cost of very deep gluten hydrolysis (DH 42%). Bioprase and Thermoase were less invasive to gluten (DH of 14.4% and 8.85%, respectively), but the hydrolysates obtained with their use contained 11,296 mg/kg and 10,520 mg/kg of gliadin, respectively. Against this background, the hydrolysate obtained by us in the presence of 2.5 U Bromelain per 1 g of WG looks very interesting (at DH = 8%, the allergenic fractions are only 1691 mg/kg). It seems that they could be a valuable functional addition to hypoallergenic products. 

### 2.5. The Effect of Dose and Type of Protease on the Antioxidant Potential of Obtained WGHs

**Figure 3:**
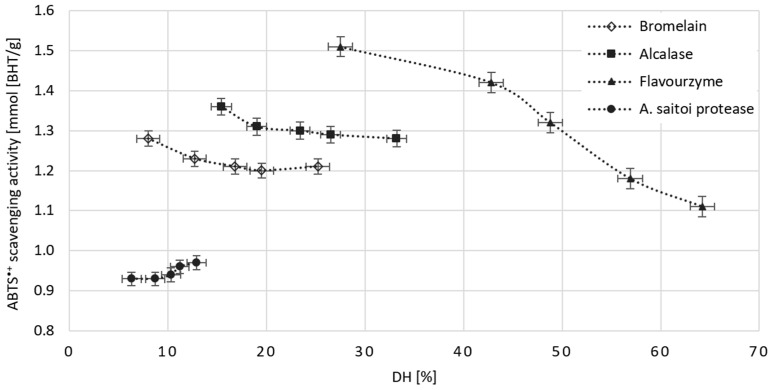
The impact of DH on the antioxidant potential of WGHs was obtained using Bromelain, Alcalase, Flavourzyme, and *A. saitoi* protease. The antioxidant potential is expressed as the equivalent amount of mmol BHT per gram of dried WGHs. All data represent the means and standard deviations (*n* = 4).

Due to its specific amino acid composition, gluten is a relatively good raw material for producing hydrolysates with high antioxidant potential. However, it requires precisely targeted hydrolysis, which is why the appropriate selection of protease is important. In this study, we examined the suitability of the following preparations for such hydrolysis: Alcalase, Bromelain, Flavourzyme, and *A. saitoi* protease. We estimated the antioxidant potential of the obtained WGHs based on their activity in scavenging the ABTS^•+^ and expressed it as a BHT equivalent (Figure 3). 

BHT is the most commonly used, highly effective synthetic stabilizer of fatty products. However, due to its negative impact on health, its use is legally limited, and the permissible dose is 100 mg/kg of fat, i.e., 0.46 mmol. We assumed that safe substitutes for such antioxidants as BHT can be sought among WGHs. Due to direct absorption from the gastrointestinal tract and the possibility of passing unchanged into the blood [32], WGHs could additionally support the natural human protection system against oxidative stress. The antioxidant potential of one gram of the dry mass of the obtained hydrolysates ranged from 0.92 to 1.51 mmol BHT. This means that when these hydrolysates are added to high-fat products in amounts of 0.3–0.5 g per kg of fat, they will be equivalent to the permissible dose of BHT. The highest antioxidant activity was exhibited by the hydrolysate obtained with the smallest dose of Flavourzyme. Its degree of hydrolysis (DH) was 27.5%. At this DH, the product is dominated by short peptides, and further depolymerization increases the proportion of free amino acids [23], which have lower antioxidant potential [7]. This is likely why WGHs obtained with higher doses of Flavourzyme had significantly lower ABTS^•+^ quenching ability. The reason why the most antioxidant-active WGHs generated by Alcalase or Bromelain also occurred at the lowest dose will be entirely different. In this case, it was likely not the length of the released peptides but their composition at the C- and N-terminal positions that were decisive. Indeed, only in the initial stage of hydrolysis will the amino acids to which the protease has the highest affinity predominate at these positions. Our results indicate that in the case of Alcalase, but also Bromelain, these are amino acids that provide relatively high antioxidant power to the peptides formed during hydrolysis. In the study by Liu et al. [23], after using Alcalase and Protex for gluten hydrolysis, five peptides (LY, PY, YQ, RGGY, and APSY) with the strongest ABTS^•+^ radical quenching activity were identified. All the identified peptides contained tyrosine at the terminal positions. On the other hand, Koo et al. [25], by analyzing the composition of WGHs produced using Alcalase and Flavourzyme after 1 and 24 h of reaction, demonstrated that with the progress of hydrolysis, the proportion of tyrosine (Y) in the resulting peptides decreased from 1.34% to 0.55% and from 1.45% to 0.46%, respectively. In the case of Flavourzyme, but not Alcalase, after 24 h of reaction, free tyrosine also appeared and constituted approximately 5% of the pool of all free amino acids. This explains the observed changes in antioxidant potential in our studies with increasing degree of hydrolysis of WGHs produced under the influence of Alcalase and Flavourzyme.

### 2.6. The Effect of Dose and Type of Protease on the Ability of Obtained WGHs to Inhibit ACE

**Figure 4:**
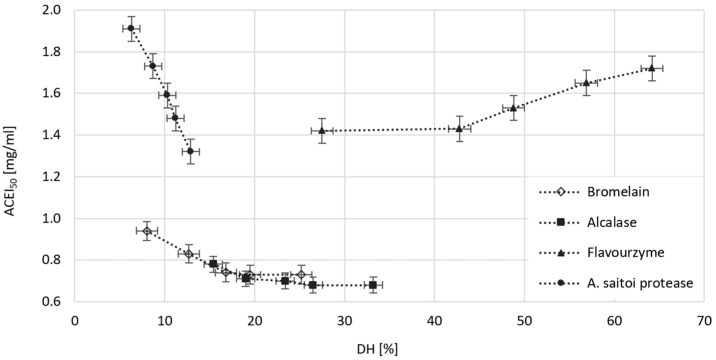
The impact of DH on the ACE inhibitory capacity of WGHs obtained using Bromelain, Alcalase, Flavourzyme, and *A. saitoi* protease. ACEI_50_—concentration (mg/mL) of WGHs inhibiting the activity of angiotensin-converting enzyme by 50%. All data represent the means and standard deviations (*n* = 4).

Angiotensin-converting enzyme (ACE) is an exopeptidase that increases blood pressure by converting angiotensin I and bradykinin [23]. This enzyme can be effectively inhibited by specific peptides, which, as a component of a food product or nutraceuticals, may have functional significance in both the prevention and treatment of hypertension [7]. The ability of WGHs obtained through the catalytic action of Alcalase, Bromelain, Flavourzyme, and *A. saitoi* protease to inhibit ACE was determined in vitro using a spectrophotometric method. The results, expressed as ACEI_50_, i.e., the concentration of hydrolysates (mg/mL) that inhibits ACE activity by 50%, are presented in Figure 4.

The effectiveness of ACE inhibition by peptides is influenced by their composition, which results from the substrate specificity of the proteases used, as well as the chain length, represented by the degree of hydrolysis. The most effective preparations for releasing ACE-inhibitory peptides were Alcalase and Bromelain. The ACEI_50_ of the hydrolysates obtained in their presence ranged from 0.8 mg/mL at the lowest enzyme doses and the lowest DH to 0.7 mg/mL at the highest doses and DH. Higher ACEI_50_ values, but also a stronger positive response to increased degrees of hydrolysis, were observed in WGHs obtained using *A. saitoi* protease. These hydrolysates provided a 50% reduction in ACE activity at concentrations ranging from 2.9 to 0.9 mg/mL. A completely different relationship was observed for WGHs obtained with the involvement of Flavourzyme. The ACEI_50_ value increased with DH from 1.42 to 1.72 mg/mL, indicating that excessive hydrolysis (49–64%) and, consequently, a significant proportion of free amino acids reduce the effectiveness of ACE inhibition. Liu et al. [23] identified dipeptide LY and tripeptide LVS as the most effective components of WGHs produced using a combination of Alcalase and Protex, both having an ACEI_50_ of approximately 0.44 mg/mL. In contrast, the most effective tetrapeptides, APSY and RGGY, inhibited ACE activity by 50% at a concentration of 0.7 mg/mL. On the other hand, Li and Yu [33], analyzing the mass and composition of 270 peptides with hypotensive effects, showed that similar efficacy can be exhibited by both those composed of 4 and 19 amino acids, with the key factor determining the effectiveness of peptides in inhibiting ACE being the C-terminal sequence. The most likely C-terminal amino acids of ACE inhibitors are Y, P, W, F, and L, while the N-terminal amino acids are R, Y, G, V, A, and I. The Alcalase used in our study showed the highest affinity for bonds with Y and L at the P1 position, while Bromelain targets bonds with G and A at the P2 position [7,9]. This means that Alcalase, especially in the initial phase of hydrolysis, primarily releases peptides with Y and L at the C-terminus, whereas Bromelain releases peptides with A and G at the N-terminus. This resulted in the lowest ACEI_50_ values for the hydrolysates, which decreased only minimally with increasing DH.

### 2.7. The Effect of Dose and Type of Protease on the Functional Properties of Obtained WGHs

One of the advantages of limited hydrolysis of wheat gluten is the ability to obtain WGHs with desirable functional properties for food technology, such as a high potential for forming stable foams and emulsions. These properties, of course, stem from the nature of the original protein but can be extensively modified through the quantitative and qualitative selectivity of the hydrolyzing agent. Table 3 presents the functional properties of WGHs obtained through hydrolysis with Alcalase, Bromelain, Flavourzyme, and *A. saitoi* protease. As a reference, we provide the values of foaming capacity (FC), foam stability (FS), emulsifying activity (EA), and emulsion stability (ES) determined for native wheat gluten and ovalbumin.

An aqueous 2% gluten solution, compared to a similar ovalbumin solution, had twice as low foaming capacity (197% vs. 400%). Additionally, after 30 min, it drained 10% more from the lamellar foam layer, resulting in an FS of 31% compared to 35% for ovalbumin. The hydrolysis of gluten, regardless of the protease used, significantly increased foaming properties if it did not exceed 19%. The highest foaming ability was observed for WGHs with DH of 10 and 8%, obtained using protease from *A. saitoi* and Bromelain, respectively. Their FC was higher than that of ovalbumin by 11.3% and 10%, respectively. However, only the WGH obtained using Bromelain formed a more stable foam than ovalbumin. FS of the WGH obtained using Bromelain was 17% higher than the FS of ovalbumin. Therefore, the prospect of using WGHs released by Bromelain as a substitute for ovalbumin in the production of meringues, foams, coconut macaroons, warm ice creams, and other desserts appears extremely promising. Such products could appeal to consumers, including vegans while remaining safe for individuals with gluten intolerance. According to the results presented in Figure 2, the foam obtained by whipping a 2% solution of WGH released by Bromelain contained up to 34 mg of gliadin per kg (classified as low-gluten). However, in the production of the aforementioned desserts, the foam constitutes a maximum of 50% of the final product’s mass, meaning that according to standards, such a product would be classified as gluten-free. Another significant outcome of the foamability analysis of WGHs obtained using various enzymes is the observation that both their FC and FS depend not only on the number of WG bonds hydrolyzed by a given enzyme but also on which specific bonds are hydrolyzed. It appears that the FS of hydrolysates depends more on the specificity of the proteases, i.e., the type of bond they attack, than FC. For example, with a similar DH (approximately 8%), WGHs released by Bromelain produced foams with an FS of 41%, whereas those released by the protease from *A. saitoi* had an FS of 22%, while the FC of both types of hydrolysates did not differ significantly. Similar conclusions can be drawn from the results obtained by the team [34] during the depolymerization of WG using pepsin and trypsin. In the cited study, WGHs with a DH of 6%, regardless of the type of protease used, showed similar foaming capacity at pH 7.0, but the stability of these foams after 30 min of storage was 37% and 2%, respectively.

As shown in Table 3, the degree of gluten depolymerization and the type of hydrolyzing enzyme also influenced the emulsifying properties of the resulting products. The starting material, a 2% aqueous solution of native gluten, when compared to an ovalbumin solution, had 30% lower emulsifying activity (EA) when mixed 1:1 (*v*/*v*) with sunflower oil. However, the stability of the O/W emulsions formed by both proteins, whether stored at 8 °C or 80 °C, was comparable and close to 100%. Among the tested WGHs, those obtained with *A. saitoi* protease exhibited the highest emulsifying activity, ranging from 56% to 66%, compared to 47% for native gluten and 67% for ovalbumin. They also had the lowest DH from 6.3% to 12.9%, which, within this range, was found to positively correlate with EA. The resulting emulsions also exhibited full stability when stored for 30 days at 8 °C and very high stability, up to 95%, after 30 min of incubation at 80 °C. Emulsifying activity at the level of 56% and 54%, significantly higher than that of the WG solution, was also exhibited by hydrolysates obtained with the smallest doses of Bromelain, characterized by DH of 8% and 12.7%, respectively. Nevertheless, only the first hydrolysate (with DH = 8%) resulted in an emulsion that was fully stable at both 8 °C and 80 °C. It should be noted that this emulsion, due to the minimal presence of allergenic sequences contributed by WGH (17 mg/kg), can be classified as a gluten-free product, in contrast to emulsions obtained from WGHs released by the protease from *A. saitoi* (116 mg/kg). Hydrolysates obtained using the other tested enzymes, namely Alcalase and Flavourzyme, exhibited only low and very low EA and ES, likely due to their overly advanced hydrolysis (the lowest DH was 15% and 28%, respectively). Other authors have already pointed out the exceptionally drastic deterioration in the emulsifying properties of WGHs obtained with Alcalase as hydrolysis progresses. According to Joye and McClements [35], only with a DH not exceeding 3% do these hydrolysates exhibit satisfactory EA, but even then, the emulsions they form are not fully stable. On the other hand, Kong et al. [14] claim that the highest EA and ES for Alcalase WGHs can be achieved with a DH of 10% and 5%, respectively. Regardless of which team is correct, the conclusion is the same—Alcalase is not a good choice if we want to obtain hydrolysates with emulsifying activity. The problem is not only the necessary minimum degree of hydrolysis but also that it involves retaining most of the allergenic WG sequences. Our calculations, based on Equation (5) y = −0.3479x^3^ + 47.907x^2^ − 2079.3x + 31,260 (R² = 0.998) describe the dependence of gliadin content in WGHs on their DH (Figure 2), indicating that the allergenic sequence content in an emulsion prepared based on Alcalase WGHs with a DH of 5% would be around 220 mg/mL, significantly higher than for stable emulsions obtained from hydrolysates produced by Bromelain or even *A. saitoi* protease.

In summary, by comparing the results regarding the emulsifying properties of the obtained WGHs (Table 3) with the observations of other researchers, it can be concluded that the key to obtaining WGHs with improved EA, and especially ES, is the substrate specificity of the enzyme. Here, the particular ability of Flavourzyme to perform very deep hydrolysis, down to di- and tripeptides, prevents success. Similarly, the unique competence of Alcalase to selectively attack hydrophobic protein sequences, thereby releasing peptides without long nonpolar blocks, significantly hinders the process. According to Pan et al. [36], it is the high surface hydrophobicity of the protein, resulting in an increased proportion of α-helix, that enhances its affinity for the surface of fat droplets and leads to more stable emulsion.

## 3. Materials and Methods

### 3.1. Materials

Wheat gluten (WG) with a protein content of 70.3%, carbohydrates of 13.3%, fiber of 5.2%, fats of 3.8%, and moisture of 7.3% was purchased from Provita (Sviadnov, Czech Republic). Proteolytic preparations: Alcalase^®^ from *Bacillus licheniformis*, Bromelain from pineapple stem, Flavourzyme from *Aspergillus oryzae*, and protease from *Aspergillus saitoi*, as well as reagents: ovalbumin, OPA (phthaldialdehyde), BHT butylhydroxytoluene, ABTS (2,2′-azinobis(3-ethylbenzothiazoline-6-sulfonic acid)), FAPGG (N-[3-(2-furyl)acryloyl]-L-phenylalanyl-glycyl-glycine), ACE (angiotensin-converting enzyme) with activity ≥2.0 U/mg protein, and Nessler’s reagent, were purchased from Sigma-Aldrich (Steinheim, Germany).

### 3.2. Determination of the Optimal pH for Gluten Hydrolysis by the Tested Proteases

The activity of the preparations against gluten was assessed at a temperature of 37 °C and at pH levels ranging from 2.0 to 9.0. To determine the activity, enzymatic preparations were used at an E:S ratio of 1:250 (*w*/*v*), with a reaction time of 10 min. The reaction was terminated by adding 5% trichloroacetic acid (TCA) in a 1:1 (*v*/*v*) ratio. The samples were centrifuged at a speed of 4000 rpm for 10 min. Next, 1 M sodium hydroxide was added to the supernatant at a ratio of 1:6 (*v*/*v*). The amount of free amino groups in the neutralized supernatant was determined using the OPA reagent [26]. For the OPA reagent, 11 mM phthaldialdehyde, 20 mM dithiothreitol, and 2.8 M methanol were dissolved in 120 mM sodium tetraborate decahydrate (adjusted to pH 9.8 with NaOH). The OPA reagent was added to the sample at a ratio of 4:1 (*v*/*v*). The absorbance of the samples was measured after 10 min at a wavelength of 340 nm. One unit (U) of enzyme activity was defined as the amount of enzyme that releases 1 µmol of amino groups per minute.

### 3.3. Determination of the Optimal Temperature for Gluten Hydrolysis by the Tested Proteases

The activity of the preparations against gluten was assessed at the optimal pH conditions for Bromelain (pH 6), Alcalase (pH 8), Flavourzyme (pH 5.5), and *A. saitoi* protease (pH 4) at temperatures of 40, 45, 50, 55, 60, 65, 70, and 75 °C. To determine the activity, enzymatic preparations were used at an E:S ratio of 1:250 (*w*/*v*), and the reaction time was 45 min. The reaction was terminated by adding 5% TCA in a 1:1 (*v*/*v*) ratio. The samples were centrifuged (4000 rpm, 10 min). The supernatants were neutralized and the amount of free amino groups was determined using the OPA reagent [26], following the procedure described in Section 3.2.

### 3.4. Preparation of Wheat Gluten Hydrolysates (WGHs)

The substrate, a 5% suspension of WG in 0.1 M citrate-phosphate buffer at the optimal pH for hydrolysis by the tested proteases, was heated to the optimal temperature for the activity of these enzymes. Subsequently, the tested proteases were introduced at E/S ratios of 2.5, 5, 10, 15, and 25 U/g of gluten, and the incubation was continued at optimal temperatures with constant stirring for an additional 240 min. After this time, the samples were heated to 100 °C and incubated for 15 min for enzyme inactivation. Simultaneously, for each experimental sample, a corresponding reference sample was prepared using thermally inactivated proteolytic preparations. Finally, the obtained hydrolysates and reference samples were centrifuged at 4000 rpm for 10 min. The supernatants were collected and stored at −20 °C for further experiments.

### 3.5. Determination of the Gluten Hydrolysis Degree (DH%)

The degree of hydrolysis of the obtained WGHs was determined using a modified method [26]. For this purpose, the appropriately diluted hydrolysates and reference samples were mixed with OPA reagent at a ratio of 1:4 (*v*/*v*). A blank sample was prepared by mixing distilled water and OPA reagent at a ratio of 1:4 (*v*/*v*). After 10 min, the absorbance of all samples relative to the blank sample was measured at λ = 340 nm. The degree of hydrolysis (HD%) was calculated using the Formula (6): DH% = (H − R) × 100/G, where H is the concentration of free amino groups in the hydrolysate (mol/L), R is the concentration of free amino groups (mol/L) in the reference sample, and G is the maximum concentration of free amino groups during total gluten hydrolysis, which is equal to 0.417 mol/L.

### 3.6. Determination of Protein Recovery (PR%)

The protein recovery in the hydrolysates was calculated based on the nitrogen content after prior sample mineralization [37]. For this purpose, 0.7 mL of GWHs, 0.14 mL of enzymatic preparation, and 50 mg of WG were combusted in a Hach apparatus with 4 mL of concentrated H_2_SO_4_ at 280 °C for 5 min. After this time, 15 mL of 30% H_2_O_2_ was added and heated for an additional 20 min. The cooled mineralized samples were adjusted with potassium hydroxide to pH 3.0 and diluted with water to 100 mL. Subsequently, nitrogen in the mineralized samples, after any necessary dilution, was determined using the Nessler method, i.e., 1 mL of 1% gum arabic and 1 mL of Nessler reagent was added to 6 mL of the sample. After 10 min, the absorbance was measured at λ = 430 nm. Protein recovery (PR) was calculated from the Formula (7): PR% = (H − E) × 100/G, where H, E, and G represent the nitrogen concentration determined by the Nessler method in the hydrolysate, enzymatic preparation, and gluten, respectively.

### 3.7. Determination of Biological Properties of WGHs

#### 3.7.1. Angiotensin Converting Enzyme Inhibitory (ACEI) Activity Assay

The ability of WGHs to inhibit ACE activity was measured using a spectrophotometric method [38]. A substrate solution consisting of 0.16 mM FAPGG, 0.01 M NaCl, and ACE solution with an activity of 0.02 U/mL was prepared in 0.1 M borate buffer at pH 8.3. Proper, reference, and control samples were prepared. Proper samples contained substrate (4 mL), hydrolysate (0.02 mL), and ACE solution (1 mL), while the control sample contained buffer at pH 8.3 (0.02 mL) instead of the hydrolysate. The concentration of dried WGH in the proper sample was 0.2 mg/mL. Proper samples and the control sample were incubated at 37 °C for 30 min, and the reactions were stopped by adding 100 mM EDTA (0.1 mL). Reference samples were prepared for each proper sample and the control sample by adding EDTA before adding the ACE solution. The absorbance of all samples was measured at a wavelength of 340 nm. The degree of angiotensin-converting enzyme inhibition by the hydrolysates was calculated using the Formula (8): ACEI% = 100 − [(R − H) × 100/(R − C)], where H, C, and R represent the absorbance of the proper samples, the control sample, and the reference samples, respectively.

#### 3.7.2. Determination of Allergenic Sequences in Gluten

Gliadin concentration in dried WGHs was determined using the ELISA immunoenzymatic test (Ridascreen^®^ gliadin competitive). The method for assessing allergenicity in gluten hydrolysates was validated in previous studies by Merz et al. [26].

#### 3.7.3. ABTS Radical Scavenging Assay

The scavenging activity of ABTS free radicals was assessed using a spectrophotometric method [23]. A solution of 7 mM ABTS and 2.45 mM potassium persulfate was prepared in 0.4 M phosphate buffer at pH 7.5. The solution was kept in darkness at room temperature for 16 h. Prior to analysis, the ABTS free radical solution was diluted with buffer to an absorbance of 0.7 at a wavelength of 734 nm. A diluted solution of ABTS free radicals (2 mL) was mixed with the hydrolysate (0.1 mL), and after 10 min, the absorbance was recorded at 734 nm (distilled water was used as a control). The final ABTS^•+^ scavenging activity (ASA) was expressed as the equivalent amount of mmol of BHT per gram of dried WGH.

### 3.8. Determination of Functional Properties of WGHs

#### 3.8.1. Foaming Capacity and Foam Stability Assay

The foaming capacity and stability of foams created by WGHs were assessed following the method described by Sadahira et al. [39] with several modifications. Aqueous suspensions of WGH lyophilizates, WG, and ovalbumins at a concentration of 20 mg/mL were dispensed in 2 mL volumes into syringes with closed ends and foamed using a blender (Sapir, SP-1163-B) for 3 min at room temperature. Foam volume was read from the scale, and the foaming capacity (FC) was calculated using the Formula (9): FC% = (A − B) × 100/B, where A is the volume after whipping (mL), and B is the volume before whipping (mL).

Subsequently, the syringe outlet was unblocked, and leakage was collected for 30 min and weighed with an accuracy of ± 0.1 mg. The foam stability (FS) was calculated using the Formula (10): FS% = A × 100/B, where A is the foam mass after 30 min (mg), and B is the initial foam mass (mg).

#### 3.8.2. Determination of Emulsifying Activity and Emulsion Stability

The emulsifying activity and emulsion stability were determined using the method described by He et al. [16], with several modifications. A water solution (20 mg/mL) of WGH lyophilizates, WG, or ovalbumin was mixed with sunflower oil in a 1:1 (*v*/*v*) ratio. Each sample was then mixed and homogenized ultrasonically at 100% amplitude in a 0.7 cycle for 2.5 min. After homogenization, the samples were centrifuged for 2.5 min at a speed of 2500 rpm. The height of the emulsified layer was measured, and the emulsifying activity (EA) was calculated according to the Formula (11): EA% = A × 100/B, where A is the height of the emulsion layer (mm), and B is the total height of the sample (mm).

Subsequently, the emulsions were shaken until the disappearance of interfacial layers and stored in a refrigerator for 30 days. After this period, the samples were centrifuged again, and the height of the emulsified layer was measured. The stability of the emulsion at low temperatures (ESLT) was determined using the Formula (12): ESLT% = A × 100/B, where A is the height of the emulsion layer after 30 days (mm), and B is the height of the emulsion layer before storage (mm).

Additionally, the emulsions were heated for 30 min in a water bath at 80 °C and then cooled to 25 °C. The cooled emulsions were centrifuged, and the height of the emulsified layer was measured. The stability of the emulsion at high temperatures (ESHT) was determined using the Formula (9): ESHT% = A × 100/B, where A is the height of the emulsion layer after heating (mm), and B is the height of the emulsion layer before heating (mm).

### 3.9. Statistical Analysis

Data were expressed as mean values ± standard deviation (*n* = 4). Statistical analysis was performed using Statistica 13.3 (StatSoft Polska). One-way ANOVA with post-hoc Bonferroni’s test was used to compare groups. In statistical analyses, a level of significance of <5% was considered significant.

## 4. Conclusions

The study demonstrated that the selection of a proteolytic enzyme is crucial for obtaining a gluten hydrolysate that combines high functional potential with maximum health benefits for the consumer and that optimizing the hydrolysis process allows for up to 92% protein recovery. It was shown that the gluten hydrolysate produced using Bromelain, with a degree of hydrolysis of 8%, forms a stable emulsion and durable foam. Moreover, the foaming activity of this hydrolysate surpasses that of egg white albumin. Additionally, this hydrolysate exhibits relatively high antioxidant activity, equivalent to 1.28 mg of BHT per gram, which can protect the fats in the final food product from peroxidation. Most importantly, food products based on such foam or emulsion would contain very low levels of allergenic sequences characteristic of wheat gluten and could be classified as gluten-free, which is unattainable with the other tested enzyme preparations. Furthermore, it was observed that Flavourzyme, even at low doses, deeply hydrolyzes gluten (at 2.5 U/g, DH = 28%), releasing amphiphilic peptides. The gluten hydrolysate with a degree of hydrolysis of 28% exhibited very high antioxidant activity (1 g equivalent to 1.52 mmol BHT), indicating that it could serve as an alternative to synthetic fat stabilizers. It was also shown that gluten hydrolysates obtained using Alcalase, with a degree of hydrolysis above 25%, could be a valuable food additive with hypotensive effects, as they effectively inhibit angiotensin-converting enzyme (ACEI_50_ = 0.68 mg/mL).

## Figures and Tables

**Table 1:** Maximum activity of Bromelain, Alcalase, Flavourzyme, and *A. saitoi* protease against WG.

Proteolytic Preparation	Optimal pH/T	Activity	
Bromelain	6.0/60 °C	188.7 ± 9.7 ^b^ U/g	3145 ± 162 ^b^ nkat/g
Alcalase	8.0/55 °C	224.2 ± 4.8 ^c^ U /mL	3737 ± 80 ^c^ nkat/mL
Flavourzyme	5.5/50 °C	137.2 ± 5.1 ^a^ U/mL	2286 ± 85 ^a^ nkat/mL
*A. saitoi* protease	4.0/55 °C	125.3 ± 7.7 ^a^ U/g	2088 ± 128 ^a^ nkat/g

Data are mean values for *n* = 4 ± SD. Column values marked with different letters are significantly different (Bonferroni post-hoc test, *p* < 0.05).

**Table 2:** Degree of hydrolysis (DH) and protein recovery (PR) in WGHs obtained under the influence of Bromelain, Alcalase, Flavourzyme, and *A. saitoi* protease.

Enzyme (t_h_, pH, T)	Bromelain (4 h, 6.0, 60 °C)					Alcalase (4 h, 8.0, 55 °C)				
E/S [U/g]	2.5	5	10	15	25	2.5	5	10	15	25
DH [%]	8.0 ± 0.6 ^a^	12.7 ± 2.1 ^b^	16.8 ± 1.3 ^c^	19.5 ± 0.2 ^d^	25.2 ± 1.2 ^e^	15.4 ± 0.4 ^a^	19.0 ± 0.4 ^b^	23.4 ± 0.6 ^c^	26.5 ± 0.4 ^d^	33.2 ± 0.6 ^e^
PR [%]	81.3 ± 3.4 ^a^	86.5 ± 1.5 ^ab^	86.5 ± 0.7 ^b^	89.6 ± 1.7 ^bc^	92.1 ± 1.6 ^c^	81.2 ± 1.9 ^a^	83.5 ± 1.9 ^ab^	85.5 ± 1.7 ^b^	89.5 ± 2.2 ^c^	91.8 ± 2.1 ^c^
Enzyme (t_h_, pH, T)	Flavourzyme (4 h, 5.5, 50 °C)					*A. saitoi* Protease (4 h, 4.0, 55 °C)				
E/S [U/g]	2.5	5	10	15	25	2.5	5	10	15	25
DH [%]	27.5 ± 1.6 ^a^	40.8± 2.2 ^b^	48.8 ± 1.8 ^c^	56.9 ± 2.4 ^d^	64.2 ± 1.3 ^e^	6.3 ± 0.7 ^a^	8.7 ± 0.3 ^b^	10.3 ± 0.1 ^bc^	11.2 ± 0.2 ^c^	12.9 ± 0.6 ^d^
PR [%]	81.4 ± 0.7 ^a^	86.7 ± 2.3 ^b^	91.1 ± 1.4 ^c^	92.8 ± 0.8 ^c^	92.2 ± 1.4 ^c^	50.9 ± 1.4 ^a^	59.8 ± 1.3 ^b^	67.7 ± 2.4 ^c^	72.6 ± 2.4 ^d^	71.1 ± 1.3 ^d^

Data are mean values for *n* = 4 ± SD. Row values for a given proteolytic preparation marked with different letters are significantly different (Bonferroni post-hoc test, *p* < 0.05). t_h_—time of hydrolysis.

**Table 3:** Foaming capacity (FC), foam stability (FS), emulsifying activity (EA), and emulsion stability at low and high temperatures (ESLT, ESHT) of WGHs obtained under the influence of Bromelain, Alcalase, Flavourzyme, and *A. saitoi* protease.

Preparation	WGHs of Bromelain					WGHs of Alcalase					Gluten
E/S [U/g]	2.5	5	10	15	25	2.5	5	10	15	25	
FC [%]	440 ± 22 ^d^	300 ± 16 ^c^	260 ± 11 ^b^	200 ± 20 ^a^	198 ± 13 ^a^	300 ± 26 ^c^	268± 17 ^c^	200 ± 14 ^b^	112 ± 17 ^a^	85 ± 12 ^a^	197 ± 15
FS [%]	41 ± 2 ^c^	31 ± 3 ^b^	28 ± 2 ^b^	22 ± 2 ^a^	23 ± 2 ^a^	13 ± 1 ^b^	11± 2 ^ab^	9 ± 1 ^a^	8 ± 2 ^a^	8 ± 2 ^a^	31 ± 2
EA [%]	56 ± 2 ^c^	54 ± 2 ^bc^	51 ± 2 ^ab^	50 ± 2 ^a^	49 ± 2 ^a^	45 ± 2 ^d^	31± 2 ^c^	21 ± 1 ^b^	8 ± 2 ^a^	7 ± 3 ^a^	47 ± 2
ESLT [%]	102 ± 4 ^c^	90 ± 4 ^b^	89 ± 3 ^b^	92 ± 4 ^b^	81 ± 3 ^a^	51 ± 5 ^c^	24± 7 ^b^	10 ± 5 ^a^	9 ± 4 ^a^	7 ± 4 ^a^	102 ± 4
ESHT [%]	95 ± 7 ^c^	69 ± 7 ^b^	36 ± 5 ^a^	33 ± 6 ^a^	30 ± 5 ^a^	25 ± 4 ^b^	23± 7 ^b^	9 ± 6 ^a^	7 ± 4 ^a^	6 ± 4 ^a^	92 ± 4
Preparation	WGHs of Flavourzyme					WGHs of *A. saitoi* protease					Ovalbumin
E/S [U/g]	2.5	5	10	15	25	2.5	5	10	15	25	
FC [%]	117 ± 26 ^c^	83 ± 17 ^bc^	65 ± 1 ^b^	70 ± 10 ^b^	23 ± 13 ^a^	325 ± 19 ^a^	402 ± 20 ^b^	445 ± 21 ^b^	342 ± 16 ^a^	340 ± 11 ^a^	400 ± 17
FS [%]	14 ± 2 ^b^	11 ± 2 ^ab^	11 ± 2 ^ab^	10 ± 2 ^a^	10 ± 1 ^a^	13 ± 1 ^a^	22 ± 2 ^b^	27 ± 2 ^c^	20 ± 2 ^b^	21 ± 1 ^b^	35 ± 2
EA [%]	43 ± 2 ^b^	8 ± 2 ^a^	7 ± 3 ^a^	5 ± 3 ^a^	5 ± 3 ^a^	56 ± 2 ^a^	60 ± 2 ^b^	60 ± 2 ^b^	65 ± 3 ^bc^	66 ± 2 ^c^	67 ± 2
ESLT [%]	65 ± 4 ^a^	24 ± 6 ^b^	8 ± 3 ^a^	7 ± 3 ^a^	5 ± 3 ^a^	99 ± 6 ^a^	101 ± 7 ^ab^	101 ± 5 ^ab^	100 ± 4 ^b^	100 ± 4 ^ab^	99 ± 3
ESHT [%]	66 ± 8 ^c^	34 ± 5 ^b^	7 ± 3 ^a^	6 ± 3 ^a^	5 ± 3 ^a^	58 ± 9 ^a^	93 ± 4 ^b^	94 ± 3 ^b^	94 ± 3 ^b^	95 ± 4 ^b^	102 ± 7

The concentration of WGHs, ovalbumin, and gluten was 2% in foams and 1% in emulsions. FS after 30 min at 21 °C, ESLT after 30 days at 8 °C, ESHT after 30 min at 80 °C. Data are mean values for *n* = 4 ± SD. Row values for a given proteolytic preparation marked with different letters are significantly different (Bonferroni post hoc test, *p* < 0.05).

## Data Availability

The data presented in this study are available on request from the corresponding author.
